# A review of the infection-associated cancers in North African countries

**DOI:** 10.1186/s13027-016-0083-8

**Published:** 2016-08-10

**Authors:** Wafaa Mohamed Hussein, Wagida A. Anwar, Mohammed Attaleb, Loubna Mazini, Asta Försti, Roxana-Delia Trimbitas, Meriem Khyatti

**Affiliations:** 1 Department of Community, Environmental and Occupational Medicine, Faculty of Medicine, Ain ShamsUniversity, Cairo, Egypt; 2 Biology and Medical Research Unit, National Center of Energy, Sciences and Nuclear Technics, Rabat, Morocco; 3 Laboratory of Onco-virology, Institut Pasteur du Maroc, Casablanca, Morocco; 4 Department of Molecular Genetic Epidemiology, German Cancer Research Center (DKFZ), Heidelberg, Germany; 5 Center for Primary Health Care Research, Clinical Research Center, Lund University, Malmö, Sweden

**Keywords:** Infection, Cancer, North Africa

## Abstract

Cancer is typically classified as a leading non-communicable disease; however, infectious agents, such as *Helicobacter pylori* (*H. pylori*), hepatitis B virus (HBV), hepatitis C virus (HCV) and human papilloma virus (HPV), contribute significantly to the pathogenesis of various cancers. Less developed countries, including countries of the North African (NA) region, endure the highest burden of infection-related cancers. The five most common infection-associated cancers in NA in order of incidence are bladder cancer, cervical cancer, liver cancer, stomach cancer, and nasopharyngeal carcinoma. This review aims to outline the epidemiologic pattern of infection-associated cancers in five NA countries (namely: Morocco, Algeria, Tunisia, Libya and Egypt) highlighting the similarities and differences across the region. The present study employed an initial literature review of peer-reviewed articles selected from PubMed, ScienceDirect and World Health Organization (WHO) databases based on key word searches without restriction on publication dates. Original research articles and reports written in French, as well as data from institutional reports and regional meeting abstracts were also included in this extensive review. Egypt, Libya, Tunisia, Algeria and Morocco were selected to be the focus of this review.

## Background

Cancer is typically classified as a non-communicable disease; however, infectious agents contribute significantly to cancer etiology [1]. The International Agency for Research on Cancer (IARC) has labelled some infectious agents as carcinogens, these include; *Helicobacter pylori* (*H. pylori*), hepatitis B virus (HBV), hepatitis C virus (HCV), *Opisthorchis viverrini*, *Clonorchis sinensis*, human papillomavirus (HPV), Epstein-Barr virus (EBV), human T-cell lymphotropic virus type 1 (HTLV-1), human herpes virus type 8 (HHV-8, also known as Kaposi’s sarcoma herpes virus; KSHV) and *Schistosoma haematobium (S. haematobium)* [2]. The data consistently point to an association between an infectious agent and a specific malignancy and ranges from 100 % (e.g. cervical cancer (CC) attributable to HPV) to 0.4 % (liver cancer caused by liver flukes), depending on the infectious agent, the cancer and the geographical location [3]. Persistent infection with certain types of HPV has been established as a necessary cause for CC, accounting for 100 % of these tumors. In addition to CC, oncogenic HPVs were found in approximately 88 % of anal cancers, 70 % of vaginal cancers, 50 % of penile cancers, and 43 % of vulvar cancers. Among other cancer types, HHV-8 has been found in nearly all tumors in patients with Kaposi’s sarcoma (KS). In endemic areas, all squamous cell carcinomas (SCCs) are assumed be attributed to schistosomiasis. *H. pylori* infection is consistently associated with gastric carcinoma and all nasopharyngeal carcinoma (NPC) cases are associated with EBV infection, while T-cell leukemia is rare in HTLV-1 negative subjects. Overall, 75 % to 80 % of cases of primary liver cancers are attributable to persistent infections with either HBV (50–55 %) or HCV (25–30 %) [1].

Malignant transformations of human cells that may be caused by infectious agents occur through different mechanisms of action [4]. These agents may act as direct carcinogens through the expression of oncogenes. The resulting onco-proteins can interact with cellular proteins leading to disruption of cell-cycle check-points, inhibition of apoptosis, and enhancement of cell immortalization. Infectious agents, such as HPV, HBV, HTLV-1, EBV and KSHV, act through this mechanism. Another mechanism of malignant transformation occurs as follows: chronic infection followed by chronic inflammation leads to the release of inflammatory mediators and the production of free oxygen radicals, which have direct mutagenic effects in addition to promoting tumor neo-vascularization and survival. Inflammation-induced cancers are associated with HCV, *H. pylori* and *S. haematobium*. Immune-suppression induced by human immunodeficiency virus (HIV-1), as well as KSHV and EBV, strongly increases the incidence of many other infection-associated cancers.

A systematic analysis by de Martel et al. from 2012 estimated that 16.1 % (about 2 million) of all new cancer cases reported during 2008, were directly attributable to infections; the majority of which (80 %, 1.6 million) originated in less developed countries. The proportion of infection-associated cancers ranged between 7.4 % and 22.9 % in developed and developing countries, respectively. According to the same study, in North African (NA) region the proportion was estimated at 12.7 %, with local variations between and within countries [1, 5].

Hepatitis B and C, HPV, and *H. Pylori* have been associated with about 95 % of infection-associated cancer cases. Cancer sites affected include cancers of the liver, cervix, selected head and neck cancers, and gastric cancers. These viruses differ in their contribution to cancer burden worldwide. HPV contributes equally to the cancer burden in both developed and developing countries. *H. Pylori* results in more cancer cases in developed countries, while HCV and HBV result in higher cancer rates in developing countries [6]. In NA, the five most common infection-related cancers in descending order are: bladder cancer, cervical cancer, liver cancer, stomach cancer, and NPC (Table 1). This review aims to demonstrate the influence of infectious agents on cancer pattern in NA countries and to summarize and provide up-to-date data in a region-specific manner.

**Table 1:** Total cancer incidence per 100,000 of most common infection-associated cancers in North African countries^a^: Globocan, 2012 [7]

	Morocco	Algeria	Tunisia	Libya	Egypt
Bladder (*Schistosoma* haematobium)	5.8	5.9	8.3	8.6	13.1
Cervix uteri (HPV)	14.3	8.5	4.8	9.7	2.3
Liver (HCV and HBV)	1.2	1.5	1.1	4.8	25.5
Stomach (Helicobacter pylori)	4.0	6.0	4.2	3.7	2.5
Nasopharyngeal (EBV)	2.3	3.2	2.3	231	0.3

^a^Total cancer incidence (not only the incidence attributed to infectious agents)

## Methods

The present study employed an initial literature review of peer-reviewed articles published in PubMed, ScienceDirect, and World Health Organization (WHO) databases with no restriction on publication date. Original research articles and reports written in French, as well as data from institutional reports and regional meeting abstracts were also included in this extensive review. In the articles and reports, data in the NA countries (Egypt, Libya, Tunisia, Algeria and Morocco) were considered eligible when the nature of epidemiological data, such as prevalence and incidence rates, were specified. Combinations of keywords used in the searches were: infectious agents (EBV, HCV, HBV, schistosomiasis, HPV, *H. Pylori*, HHV-8 and HTLV-1); country name (Egypt, Algeria, Morocco, Tunisia, Libya, NA and Maghreb); cancer type (bladder, cervical, liver, hepatocellular carcinoma (HCC), anogenital, and NPC).

### Schistosomiasis and bladder cancer

**Fig. 1:**
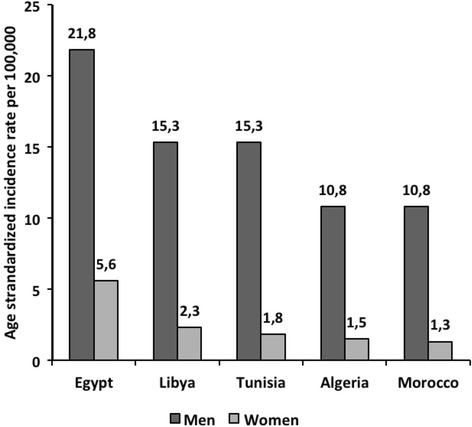
Estimated age standardized incidence for bladder cancer in North African Countries: Globocan, 2012 [7]. Estimated age standardized incidence for bladder cancer in five North African Countries according to GLOBOCAN 2012 report shows that Egypt has the highest burden of bladder cancer

Bladder cancer is the 11th most common cancer worldwide, affecting predominantly men (77 %), with an estimated 429,793 new cases in 2012 and 165,068 deaths in the same year [7, 8]. Egypt has the highest age-standardized incidence rate (ASR) of bladder cancer in men in NA (21.8/100,000 persons per year), followed by Algeria and Morocco (15.3/100,000 persons per year) and Libya and Tunisia (10.8/100,000 persons per year) (Fig. 1). Egyptian men also have the highest age standardized mortality rate in the world (11.1 per 100,000 men), which is twice the rate in Europe (5.2/100,000) and three times that in the United States (4.0/100,000) [7]. Consistent with the worldwide data, cases in men comprise a higher fraction of bladder cancer as compared to women in NA (Fig. 1). This observation is likely due to the high prevalence of urinary schistosomiasis, and the high rate of smoking among men [9].

In the 1920s *S. haematobium* infection rates in Egypt were as high as 70–80 %. As a direct result of several control strategies that have been implemented throughout the country for the last two decades, Egypt has recently reached low schistosomiasis endemicity [10]. The prevalence of schistosomiasis dropped to 6.6 % in 1993 and then to 1.9 % in 2002 and 1.2 % in 2006 [11, 12]. Schistosomiasis is mostly eliminated in both Tunisia and Morocco, as no new cases were reported in these countries since 1984 and 2004, respectively [13]. The Moroccan schistosomiasis elimination strategy started in 1994 and disease transmission was curtailed by 2009 based on the absence of serum antibodies, indicating undetectable or complete absence of transmission, in a sample of 2,382 children selected from provinces with a history of high incidence [14]. In Libya, *S. haematobium* is endemic in some limited regions (near the Mediterranean coast, in the central part and near the southwestern border of Algeria). The estimated prevalence of *Schistosoma* in Libya between 2003 and 2010 was 5 %. In Algeria, *S. haematobium* is absent in most parts of the country and the risk of infection is localized in the province of Boumerdes [15].

Regarding bladder cancer incidence, the Algerian cancer registry, Setif, reported an increased trend in bladder cancer during a study period of 25 years (from 1986 to 2010). Bladder cancer rates (per 100,000) increased in men from 1.6 in 1986–1990 to 10.3 in 2006–2010 [16]. Similarly, in Morocco, based on the Casablanca cancer population registry, the ASR of bladder cancer increased in men from 5.8 in 2004 to 10.2 in 2007 [17]. Different trends were observed in the other NA countries, with either a stabilized or a decreasing tendency. The incidence rates remained stable over time in Tunisia (1993–2006) [18]. Bladder cancer ASR decreased from 11.7 in 2003 to 3.2 in 2012 among men in Libya [19]. In Egypt, the relative frequency of bladder cancer dropped from 27.6 % in 1970 to 11.7 % in 2007, which correlates well with a decrease of Schistosoma association from 82.4 % to 55.3 % during the same study period. A significant rise of tobacco-associated transitional cell carcinoma (TCC) from 16 % to 65.8 % and a decrease in SCC from 75.9 % to 28.4 % was however reported for the same period [20].

Shistosomiasis-associated bladder cancer presents a distinctive clinicopathologic profile, different from that reported from the Western countries. In contrast to the Western countries, which predominantly exhibit TCC as the major form of bladder cancer (estimated at 90–95 %), Egypt has reported as much as 75 % of their cases as SCC in the past [9, 21, 22]. Between 1970 and 1974, 26.7 % of patients registered in the National Cancer Institute (NCI) at Cairo University suffered from bladder cancer attributed to schistosomiasis [21]. Another study of 2778 patients at the NCI, showed that bladder cancer patients in 2005 had a six fold increased risk of TCC vs. SCC compared with patients treated in 1980 [23]. Thus, despite the considerable elimination of *S. haematobium*-related infections and hence the resulting bladder cancer, the effect of that had been impeded by tobacco-related bladder cancer among Egyptian men.

### Human Papilloma virus and cervical cancer

**Fig. 2:**
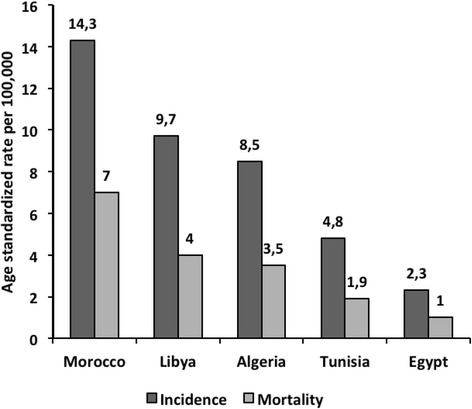
Estimated age standardized incidence and mortality rates for cervical cancer in North African countries: Globocan, 2012 [7]. Estimated age standardized incidence and mortality rates for cervical cancer in five North African countries according to GLOBOCAN 2012 report shows an increasing East-West gradient of cervical cancer

Cervical cancer is the world’s third most common cancer among women, with an ASR of 14 per 100,000 women. The main bulk of CC cases occur in developing countries (85 %), yet NA countries show an overall low incidence (ASR: 6.6 per 100,000). Despite the relatively low incidence rates, CC is the second most common cancer among women in Algeria and Morocco and the third most common cancer in Tunisia. An increasing East-West gradient exists, ranging from as low as 2.3/100,000 women in Egypt to as high as 14.3/100,000 women in Morocco (Fig. 2) [7].

The age specific rates in Algeria and Morocco, were found to increase rapidly with age until 50–54 or 55–59 years, and then remained constant (Algeria) or decline (Morocco). Incidence rates reached relatively high values (almost 60/100,000) in Morocco between the ages of 50–54 years and around 40/100,000 in Algeria for ages above 55 years. In Egypt, the incidence rate was lower than 10/100,000 across all age groups. No cases of CC were observed among women below 30 years of age in Algeria, Morocco, and Egypt [24].

Almost 100 % of all CC cases are caused by the HPV, which ranks as the most common sexually transmitted infection globally [3]. There are more than 150 strains of HPV with varying carcinogenic potential. HPV-16 and 18 overshadow other strains (31, 33, 35, 45, 52 and 58) contributing to over 70 % of all CC cases and being responsible for 41–67 % of high-grade cervical lesions and 16–32 % of low-grade cervical lesions [25–27]. Paucity of published data in the NA region obscure the true epidemiological picture, however it is estimated that HPV prevalence falls between 5–12 % in low-risk population groups and 20–49 % in high-risk groups, with an average of 21.3 % [28]. Data of a meta-analysis also reported that the crude and adjusted HPV prevalence among women with normal cytological findings in NA (Egypt, Tunisia, Morocco and Algeria) were estimated at 10.9 % and 9.2 %, respectively [29]. The average prevalence is slightly higher in cases of cervical intraepithelial neoplasia (CIN) and established CC cases (25–90 % and 61–98 %, respectively) [28]. Mirroring the global situation, HPV16 is the most prevalent type among diagnosed cancer cases in NA countries (59 %), followed by HPV-18 (8.6–17 %) [30]. According to data available till 2013, NA countries resemble each other in HPV prevalence, which hovers around 10.5 %, aside from Tunisia which has a higher national prevalence (14.6 %), reasons for which are presently unclear (Table 2) [31].

**Table 2:** HPV prevalence in North African countries among different cell types according to data available till 2013 [31]

Country	HPV prevalence in women with normal cytology	HPV 16 and/or 18 in women with			
		Normal cytology	Low grade lesions	High grade lesions	Cervical cancer
Egypt	10.5	5.1	18.5	40	78.4
Libya	10.7	5.1	18.5	40	78.4
Tunisia	14.6	5.2	18.5	40	78.4
Algeria	10.5	9	18.5	40	77.1
Morocco	10.5	2.9	20.9	40	79.2

### Human Papilloma virus and other ano-genital cancers

While HPV is the major cause of various types of ano-genital cancers, such as anal, vulvar, vaginal, and penile cancers, few studies report this correlation perhaps due to their low incidence rates, and a focus on CC, respectively. Anal cancer has a low worldwide incidence of 1 per 100,000 in the general population. Interestingly, the majority of vulvar cases were found in developed nations. Contrary to vulvar cancer, a higher prevalence (68 %) of vaginal cancer was observed in developing nations. Equally rare is worldwide incidence of penile cancer (22,000 new cases/year), which is highly positively correlated with CC [32–34]. Low incidences of ano-genital cancers have been reported across NA countries. ASRs of ano-genital cancers in NA countries did not exceed 3.1/100,000 for anal cancers, 0.7 for vulvar cancer and 0.4 for vaginal cancer (Table 3). Libya and Morocco do not currently have any official, published data [35–37].

**Table 3:** Age-standardized incidence rate (ASR) of ano-genital cancers in North African countries [35–37]

	Egypt	Tunisia	Algeria
Anal cancer	0.4	0.2	Algiers: Male 3.1; Female 2.5 Setif region: Male 0.1; Female 0.1
Vulvar cancer	0.7	0.3	Algiers: 0.1 Setif region: 0.0
Vaginal cancer	0.2	0.4	Algiers: 0.1 Setif region:0.2
Penile cancer	0.0	0.0	Algiers : 0.0 Setif region:0.0

The only available data on HPV in anogenital cancers from NA are from a population from west Algeria, reporting a prevalence of HPV of 40 % in vaginal cancers, 17 % in vulvar cancers and 33 % in anal cancers [38].

### Viral hepatitis and liver cancer

**Fig. 3:**
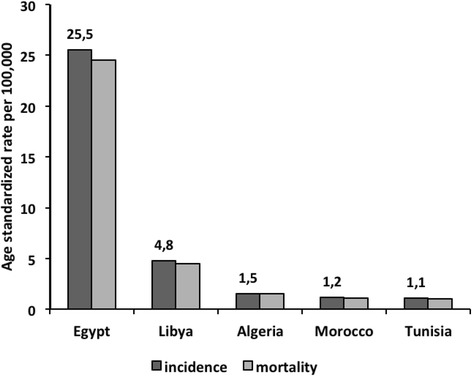
Estimated age standardized incidence and mortality rates for liver cancer (in both sexes) in North African countries: Globocan, 2012. [7]. Estimated age standardized incidence and mortality rates for liver cancer in five North African countries according to GLOBOCAN 2012 report shows that Egypt has the highest burden of liver cancer with a decreasing East-West gradient

Globally, liver cancer or HCC is the fifth cancer among men (7.5 % of all cancers in men) and the ninth among women (3.4 % of all cancers in women). About 85 % of cases occur in developing countries with 2.4:1 male to female ratio [7, 39]. In NA, liver cancer is the most common cancer in men and the second most common one among women with an estimated ASR of 18 and 7 per 100,000 men and women, respectively. A large disparity exists with a decreasing East-West gradient observed across the region, where incidence in both sexes ranges from a lowest of 1.1/100,000 in Tunisia to a highest of 25.5/100,000 in Egypt (Fig. 3). In Egypt, the male:female ratio reaches 3.8:1, probably due to the higher prevalence of HBV and HCV among men. In addition, the relatively high rates of NHL in Egypt may be explained, in part, by the high HCV prevalence in the country [7].

About 5 % of all cancers are thought to be the direct result of chronic infections with hepatitis viruses [3]. A strong geographic correlation has been found globally between the incidence of liver cancer and the prevalence of hepatitis B surface antigen (HBsAg) or hepatitis C antibody (anti-HCV) [40–43]. Chronic HCV is implicated in most HCC cases in developed countries and in one-third of cases in developing countries [44]. In NA there is a 33-fold increase in HCC with HCV infection and a 10-fold increase with HBV infection [45]. In addition, there is evidence for a causative association between chronic HCV infection and non-Hodgkin lymphoma (NHL), although the underlying mechanism is still not fully understood [46–48].

### Hepatitis B

Most NA countries have an intermediate endemicity of HBV (Table 4) [49]. In Algeria, there is a significant lack of recent data on the prevalence of HbsAg in the general population, with the last publication dating back almost 20 years ago. The WHO estimates the prevalence in Algeria to stand anywhere between 2 % to 7 %, while Khelifa et al. reported it as 2.2 % in North Eastern regions in 1998 with HBV genotype D being the most common there accounting for 93 % of all HBV infections [50].

**Table 4:** Prevalence of HBV in North African countries according to data available till 2013 [49]

Country	HBV prevalence (%) in population	HBV prevalence (%) in Hemodialysis patients	Most common genotypes	Less common genotypes
Egypt	4	4.1	D	Subtype D1
Libya	2–7	2.6 [52]	D	
Tunisia	2–7	No data	D	A, E
Algeria	3.6	No data	D	A, E
Morocco	1.3	2	D	A

The sero-prevalence of HBsAg in the general population in Libya varies between 1.3 % and 5.8 %. A national sero-epidemiological study conducted between 2004 and 2005 showed a prevalence of 2.2 % with 120,000–150,000 chronic HBsAg carriers [51]. A study on hemodialysis patients detected HBsAg prevalence of 2.6 % [52]. A low prevalence (1.3 %) was found among blood donors. HBV genotype D is the most prevalent in Libya [53].

Morocco has a low estimated prevalence for chronic HBV, ranging between 1 % and 3 % [54–58]. The HBsAg burden in the general population is 1.5 %, which was similar to that reported among health professionals (1 %), yet this figure increases to 1.7 % among sexually active subjects and to 2 % among hemodialysis patients [54–56, 59]. At Moroccan blood banks, the prevalence has remarkably decreased between 2000 and 2010 from 2.5–2.8 % to 0.8–1.3 % [57, 59, 60]. HBV genotype D accounts for 90 % of all HBV infections in Morocco [61].

In Tunisia, the estimated HBV prevalence ranges between 2 % and 7 %, being highest in South and Midwest regions. A survey on blood donors in 1994 revealed a prevalence of 5 % [62]. In 2010, the overall prevalence of HBsAg burden in a population of chronic carriers and of volunteers was 5.3 % and 2.9 % respectively. A large study conducted in 1997 found that 6.5 % of the study group was HBsAg positive [63]. HBV genotype D was found in 80 % of patients in Tunisia with dominant sub-genotype D7 [64, 65].

In Egypt, an earlier study reported HBsAg prevalence between 8 % and 11.7 % [66]. Recently, HBV prevalence in the general population has been varying from 1.3 % to 6.7 % [49]. Recent blood donor screening revealed a prevalence of HBsAg of 1.3–1.4 % [67, 68]. However, another cross-sectional study found that 16.6 % of blood donors were positive for total anti-HBV core antibodies of which 64 % were positive for HBsAg [69]. The prevalence of HBV among Egyptian liver cancer patients was 25.9 % and was shown to decrease over time [70]. HBV genotype D accounts for an estimated 87 % of the total infection in Egypt [71].

### Hepatitis C

The global HCV prevalence is 2.35 % with estimated 160 million chronically-infected people [72]. It is a major cause of severe liver disease resulting in 0.35 million annual deaths [73]. HCV infection is a major public health issue in the NA Maghreb region (Morocco, Mauritania, Algeria, Tunisia, and Libya), where the prevalence ranges between 1–2 %. The highest prevalence however is reported in Egypt, ranging from 6 % to 28 % with an average of 14.7 % [74]. In addition, immigrants from the NA region to Europe were shown to have a higher prevalence compared to the general European Union population in host countries, raising the issue of national immigrant screening programs [75–77].

In Algeria, a survey conducted in 1995 among blood donors and pregnant women, found an HCV prevalence of 0.2 % [78]. The Algerian Ministry of Health estimated that the prevalence had reached 2.5 % in 2009 [79]. High prevalence rates, were found among hemodialysis patients (25 % to 53 %) and in hemophiliacs (31.6 %) [80]. In Algeria, subtype 1b is the most prevalent genotype (86.2 %) [81].

In the mid-1990s, the prevalence of HCV cases among Libyan general population was 8 % and was labeled as ‘community acquired’ [82]. A more recent national sero-epidemiological survey denoted that the prevalence had declined to 1.2 % [51]. Prevalence was higher among renal dialysis (20.5 %) and poly-transfused patients (11 %), transmission being mainly nosocomial [83]. Genotype 4 was the most common (35.7 %), followed by genotype 1 (32.6 %) [84].

In Morocco, the estimated prevalence among the general population and blood donors was 0.3 % and 2 % respectively [85–87]. The highest prevalence rates were observed in hemodialysis patients (34 %-68 %), hemophiliac patients (42 %), and intravenous drug users (60.1 %) [85, 88–91]. A survey conducted in 2001 in Casablanca revealed a 5 % prevalence of anti-HCV antibodies in traditional barbers. A more recent study showed a low prevalence among young army recruits (0.35 %), blood donors (0.33 %), and hospitalized patients (3.08 %) [60]. The most commonly found HCV genotypes were 1b (47.6 %), 2a/2c (37.1 %) and 1a (2.8 %) respectively [92].

In Tunisia, the average prevalence of anti-HCV antibodies is 1.6 % ranging between 0.3 % in the south and 3 % in the northwest region where several reports revealed the presence of a chronic hepatitis C cluster. An average prevalence of 0.6 % was reported in blood donors. Genotype 1b predominates and the prevalence ranges from 79 % to 90 % [62, 93–95].

The Egyptian Demographic Health Survey of 2008 estimated the prevalence of anti-HCV antibodies to be 14.7 %; the highest in the world to date. This inflated prevalence is thought to be associated with the mass parenteral anti-schistosomiasis therapy implemented during the 1950s-1980s [96–98] resulting in a large reservoir of chronic HCV. Between 2000 and 2007 the annual sero-prevalence of HCV among blood donors declined from 17.7 % to 7.4 % [67]. However, ongoing endemic transmission is suggested with an estimated incidence of nearly 6.9/1,000 persons per year [96, 99]. HCV genotype G4 is the most common genotype in Egypt [98].

A meta-analysis of 32 case-control studies reported that co-infection with HBV and HCV is strongly associated with HCC (OR = 165), compared with mono-infection with HCV (OR =17) or HBV (OR = 23) [100]. Similarly, a recent study in the NA region reported a 33-fold increase in HCC with HCV infection and a 10-fold increase with HBV infection [ 45]. This risk increased 84-fold when patients were co-infected by both viruses, suggesting a synergistic effect between the two infections, although the exact mechanism has not yet been established.

A multicenter case-control study conducted between 2002 and 2005 in patients with HCC from Algeria, Morocco and Tunisia, reported that 60 % of HCC patients were positive for anti-HCV and 18 % for HBsAg [45]. In Egypt, there was a significant increase in the annual proportion of patients with HCC among chronic liver disease patients attending the Cairo liver center, during a study period ranging from 4.0 % in 1993 to 7.2 % in 2002, with a significant drop of HBsAg positivity among HCC patients from 38.6 % to 20.5 % and a slight increase of HCV positivity from 85.6 % to 87.9 % [101].

The Algerian Cancer Registry in Setif was the only one that could provide longitudinal data on HCC over a 25-year period (from 1986 to 2010). The liver cancer rate was found to increase since 1996–2000 after a decrease for both sexes (annual percent changes of +8.9 % in men and +5.4 % in women) [16]. In Libya, no significant change in liver cancer incidence was observed between 2003 (ASR = 3.3) and 2012 (ASR = 4)) [19].

### *H. Pylori* and stomach cancer

**Fig. 4:**
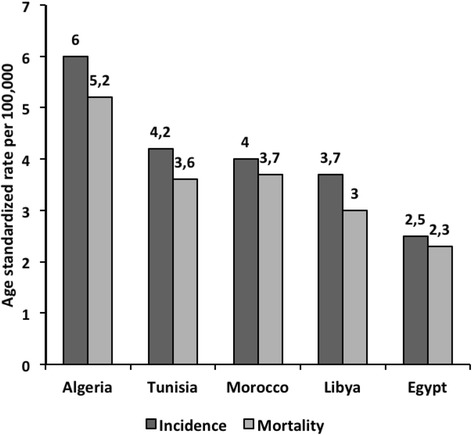
Estimated age standardized incidence and mortality rates for stomach cancer (in both sexes) in North African countries: Globocan, 2012 [7]. Estimated age standardized incidence and mortality rates for stomach cancer in five North African countries according to GLOBOCAN 2012 report shows that Algeria has the highest burden of stomach cancer

Stomach cancer is the 4th common cancer in men and the 6th in women worldwide with an ASR of 17.4 and 7.5 per 100,000 respectively. Stomach cancer incidence is much less in NA (ASR: 4.3 and 2.7 per 100,000 in men and women, respectively) with an increasing East- West gradient (Fig. 4) [7].

*H. pylori* infection is the etiological agent in various gastric pathologies and the disease severity has been attributed to some *H. pylori* genotypes [102]. It is estimated that *H. pylori* infection increases the risk of stomach cancer by nearly six times. Prevalence rates differ by age, ethnicity and socioeconomic characteristics, being higher in developing countries [103].

In Morocco, a 69 % prevalence of *H. pylori* infection was found among patients with digestive complaints. Commonly infected sites were the atrium (73 %), the corpus (21 %) and the pylorus (6 %). The potentially carcinogenic cagA antigen was detected in 42.3 % of cases [104]. In Egypt, several studies showed high prevalence of infection in various settings. *H. pylori* IgG antibodies were detected in 68 % of a group of women with primary fibromyalgia [105]. *H. pylori* prevalence of 72.38 % was found among school children [106]. In Tunisia, 66.7 % of a group of symptomatic patients were seropositive for *H. pylori* compared to 64 % of a group of blood-donors [107]. The prevalence of *H. pylori* infection reached 51.4 % among six-year old children [108]. The estimated prevalence of *H. pylori* infection in Algeria was over 70 % [109]. In 1989, a sero-epidemiological study found that the prevalence among children in the first decade of life was 45 % which rose steadily to reach 92 % among those in the fifth decade [110]. Despite the high infection rates in NA countries, stomach cancer remains relatively low, indicating that other determinants such as genetic factors or virulence of the organism influence the carcinogenic potential and ultimate cancer development.

### Epstein-Barr virus associated cancers

NPC is the 22nd most common cancer worldwide, but it is the 6th most common cancer in NA. In addition to NA it is prevalent in Southern China, Southeast Asia, Japan and the Middle East [111, 112]. EBV is a group I carcinogen, implicated in NPC, Burkitt’s Lymphoma, Hodgkin’s, non-Hodgkin’s and T cell lymphoma [113].

**Fig. 5:**
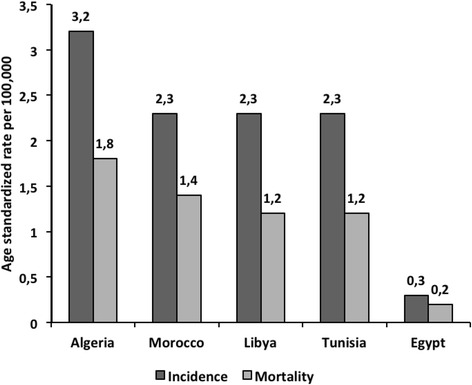
Estimated age standardized incidence and mortality rates for NPC (in both sexes) in North African Countries: Globocan, 2012 [7]. Estimated age standardized incidence and mortality rates for NPC in five North African countries according to GLOBOCAN 2012 report shows highest levels of NPC in Algeria

EBV infects over 95 % of the global adult population [114], yet NPC results from an interaction between EBV infection and environmental/genetic factors [112]. The highest observed incidence of NPC in NA is in Algeria (3.2/100,000) and the lowest is in Egypt with 0.3/100,000 (Fig. 5). Interestingly, some typical NA foods seem to be associated with increased risk of NPC [115, 116]. A study conducted over 4 years (2002–2005), in Morocco, Algeria and Tunisia reported that the consumption of butyric acid (found in rancid butter (*smen*), rancid sheep fat, and dried mutton stored in oil (*quaddid*)) was significantly associated with an increased risk of NPC, with the presence of butyric acid in these traditional foods acting as a potential EBV activator [116].

### HHV8 and Kaposi’s sarcoma

Kaposi’s sarcoma (KS) does not appear in the top 25 cancers in the world, however, it is the 10th most common cancer in Africa, with an ASR of 4.7/100,000 probably due to the high prevalence of HIV [7]. In Morocco, anti-HHV8 antibody was found in 92 % of KS cases, mostly affecting males [117, 118]. Kaposi sarcoma was the cause of death in 6 % of a sample of HIV-positive patients [119].

In Tunisia, the sero-prevalence of HHV8 was 13.8 % in blood donors, 13 % in pregnant women, and 12 % in children [120]. HHV8 was found in 17 % of kidney transplant patients [121]. Another study noted that 7 % of adult renal transplant recipients developed various cancers, of which 41.6 % developed KS [122]. Kaposi sarcoma was found in 4.5 % of skin cancer patients registered in southern Tunisia over a period of 24 years (1979–2002) [123]. In a small cohort with acquired immunodeficiency syndrome (AIDS), KS emerged in 22.7 % of cases within 0 to 10 years. This time frame was similar in the neighboring Morocco [124]. In Egypt, a prospective study on 1–4 year old children with febrile syndrome showed that 41.9 % were seropositive for HHV-8 [125]. There is a general lack of data on this subject in Algeria.

### HTLV-1 and T-cell Leukemia

There is a close association between T-cell Leukemia and the presence of antibodies to HTLV-I [126]. However, few studies have been carried out in the last decade in Africa and the number of tested individuals is limited. Sero-positivity for HTLV-I in Morocco is 0.6 %. In Tunisia, Algeria and Egypt, HTLV-I/II infections were not observed in blood donors or recipients [127].

## Conclusion

Differences in the rates of infection-related cancers across NA countries are manifested in the high incidence of bladder and liver cancers and NHL in Egypt versus the remarkably increasing East-West gradient of CC. Rates of NPC were also high in all the NA countries except Egypt.

The burden of infection-related cancers is still underestimated worldwide and associations with new infectious agents remain yet to be explored. If infectious diseases associated with cancers were prevented, it is estimated that there would be 26.3 % fewer cancer cases in developing countries and 7.7 % in developed countries.

The administration of the HBV vaccine in addition to the routine screening of blood and blood products for HBV and HVC have resulted in a reduced incidence of liver cancer. Furthermore, HPV vaccines which protect against HPV-16 & 18 are now available and have the potential to reduce the incidence of CC. A paradox is the incidence of SCC of bladder cancer, which markedly decreased in Egypt as a result of the success of schistosomiasis control, although increased smoking doubtless is a factor contributing to the increased proportion of TCC bladder cancer in Egypt. The intermediate prevalence of many communicable diseases in NA requires a constant epidemiological surveillance and the rapid adoption of available preventative vaccines in addition to health education about region-specific risk factors. Therefore the strategy should shift from a therapy-based one which is employed after the development of a specific malignancy is linked to an infectious agent to a preventative one based on national vaccination campaigns, population education on risk factors and other lifestyle-related preventative measures.

## Abbreviations

AIDS, Acquired immunodeficiency syndrome; ASR, Age standardized incidence rate; CC, Cervical cancer; EBV, Epstein-Barr virus; *H. pylori*, *Helicobacter pylori*; HBsAg, Hepatitis B surface antigen; HBV, Hepatitis B Virus; HCC, Hepatocellular carcinoma; HCV, Hepatitis C Virus; HHV-8, Human herpes virus type 8; HIV-1, Human immunodeficiency virus; HPV, Human papillomavirus; HTLV-1, Human T-cell lymphotropic virus type 1; IARC, International Agency for Research on Cancer; IgG, Immunoglobulin G; KS, Kaposi’s sarcoma; KSHV, Kaposi sarcoma- associated herpes virus; NA countries, North African countries (Egypt, Libya, Tunisia, Algeria and Morocco); NA, North Africa; NCI, National Cancer Institute at Cairo University; NHL, Non-Hodgkin lymphoma; NPC, Nasopharyngeal carcinoma; OR, Odds ratio; *S. haematobium*, *Schistisoma haematobium*; SCC, Squamous cell carcinoma; TCC, Transitional cell carcinoma; WHO, World Health Organization
